# A novel frameshift *STAG1* variant exhibiting haploinsufficiency due to the nonsense-mediated mRNA decay: a case report and literature review

**DOI:** 10.3389/fped.2025.1648430

**Published:** 2025-10-24

**Authors:** Cuicui Jiang, Ke Wu, Ying Zhou

**Affiliations:** ^1^ Obstetrics Department, Quzhou Maternal and Child Health Care Hospital, Quzhou, Zhejiang, China; ^2^ Laboratory of Prenatal Diagnosis Center, Quzhou Maternal and Child Health Care Hospital, Quzhou, Zhejiang, China

**Keywords:** intellectual disability, *STAG1* gene, haploinsufficiency, frameshift, functional studies

## Abstract

**Background:**

The heterozygous *STAG1* gene (OMIM*604358) variants are associated with autosomal dominant intellectual developmental disorder 47, known as mental retardation autosomal dominant 47 (MRD47, OMIM#617635). Although more than 10 *STAG1* variants have been reported, functional studies *in vitro* have not been performed. Our functional studies of a novel frameshift *STAG1* variant in a Chinese boy have provided preliminary evidence confirming that the underlying pathogenic mechanism of MRD47 may be associated with *STAG1* haploinsufficiency.

**Methods:**

Trio-based whole-exome sequencing (trio-WES) was performed on genomic DNA (gDNA) of peripheral blood samples from the boy and his parents. Mutant *STAG1* expression vectors pcDNA3.1(+)-FLAG-STAG1-mut and control pcDNA3.1(+)-FLAG-STAG1-WT mammalian expression vectors were constructed. Both vectors were transformed into HEK293T cells. The assays of relative *STAG1* gene mRNA expression and STAG1 protein expression were adopted.

**Results:**

Trio-WES identified a novel heterozygous frameshift *STAG1* gene variant (NM_005862.3) c.500dup (p.Gly168TrpfsTer13). Our *in vitro* functional findings revealed that this variant resulted in a dramatic reduction in the formation of STAG1 protein due to the decay of mutant *STAG1* mRNA. The underlying pathogenic mechanism of MRD47 may be related to *STAG1* haploinsufficiency.

**Conclusion:**

MRD47 exhibits non-specific characteristics and diverse clinical phenotypes. Our functional studies have provided preliminary evidence confirming the haploinsufficiency of the *STAG1* gene as the underlying pathogenic mechanism of MRD47. This study also expanded the mutational spectrum of the *STAG1* gene and the clinical spectrum of MRD47.

## Introduction

Autosomal dominant intellectual developmental disorder 47, alias mental retardation autosomal dominant 47 (MRD47, OMIM #617635) is caused by heterozygous variants in the *STAG1* gene (OMIM*604358) on chromosome 3q22.3. The heterozygous intragenic deletions within the *STAG1* gene, *de novo* heterozygous missense and frameshift *STAG1* gene variants in 17 unrelated patients with similar phenotypes (intellectual disability/developmental delay, growth retardation, feeding difficulties, facial dysmorphism, epilepsy, autistic features) were first reported by Lehalle et al. who considered the *STAG1* gene could be a novel gene responsible for non-specific syndromic intellectual disability (1). For now, more than twenty cases with *STAG1* variations have been described in the literature, but functional studies of these variants *in vitro* have not been performed. These authors postulated that the neurodevelopmental phenotypes were caused by loss-of-function (LoF) effects of *STAG1* variants. Herein, we first reported a Chinese patient with a novel heterozygous frameshift *STAG1* variant, and performed a serial of functional studies on this variant. This study preliminarily suggested the pathogenic mechanism of MRD47 may be associated with *STAG1* haploinsufficiency.

## Materials and methods

### Clinical features

The patient was a 3-year-old boy who first presented at the age of 2 years for evaluation of developmental delay. There was no family history of congenital anomalies or intellectual disability/neurodevelopmental disorders (NDD). He was born at 38 weeks’ gestation with normal birth weight and length following an uncomplicated pregnancy. His developmental milestones were delayed; he raised his head at 5 months, sat independently at 10 months, stood up at 18 months and walked at 19 months. The boy was followed up until the age of 3 years and 10 months. At the last follow-up, he still could not say any words. His height was 92 cm (<10th centile) and weight was 12 kg (<10th centile). His occipitofrontal circumference was within the normal range. The electrocardiogram and cerebral magnetic resonance imaging were normal. His vision and hearing evaluations were both normal. Facial dysmorphisms, limb anomalies, autistic features and other behavioral anomalies have not been observed.

### Whole-exome sequencing (WES)

Trio-based whole-exome sequencing (trio-WES) was performed on genomic DNA (gDNA) of peripheral blood samples from this boy and his patients using the Blood Genome Column Medium Extraction Kit (Kangweishiji, China) according to the manufacturer's instructions. The extracted DNA samples were subjected to quality control using a Qubit 2.0 fluorimeter and electrophoresis with a 0.8% agarose gel for further protocols. The xGen™ Exome Research Panel v2 (designed by Integrated DNA Technologies) was used for WES. Quality control (QC) of the DNA library was performed using an Agilent 2100 Bioanalyzer System (Agilent, USA). DNA nanoball (DNB) preps of clinical samples were sequenced on ultra high throughput DNBSEQ-T7 platform (MGI, Shenzhen, China) with paired-end 150 nt strategy following manufacturer's protocol.

### Bioinformatic analysis

Sequencing data was analyzed according to our in-house (Chigene Translational Medicine Research Center) procedures. Raw data were processed quickly for adapter removal and low-quality read filtering, and then data quantity and data quality was statistics. The trimmed reads were then mapped to the University of California at Santa Cruz (UCSC) GRCh37/hg19 reference genome using Burrows-Wheeler Aligner (BWA) software (version 0.7.17). The Genome Analysis ToolKit (GATK) software (version 4.2.1.0) was used for single nucleotide polymorphisms/variants (SNPs/SNVs) and short insertions/deletions (indels) (<50 bp) calling. Samtools (version 1.22.1) and Picard software (version 2.22.1) packages were used to generate clean binary alignment map (BAM) data by removing duplicate data. Variants were annotated for analysis using the single nucleotide polymorphism database (dbSNP, version 5.3), gnomAD exomes database (version 4.1.0) and Chigene in-house minor allele frequency (MAF) database. Tools of pathogenicity prediction like REVELAL and AlphaMissense were used for predicting possible impact of variants. As a prioritized pathogenicity annotation to the American College of Medical Genetics and Genomics (ACMG) guidelines, Online Mendelian Inheritance in Man (OMIM), Human Gene Mutation Database (HGMD) and ClinVar databases were used as conferences of pathogenicity of every variant.

### Variants classification

As per the guidelines of ACMG for interpreting sequence variants, variants were classified. Classification considered the position of the variant in the human genome, MAF, the pathogenicity prediction of variants, disease mechanism, clinical phenotypes, literature evidence, evolutionary conservation.

### Variants verification

gDNA samples was used for the verification of this variant. All reactions were carried out in a total volume of 20 μl using the Taq DNA polymerase. After confirmation of the size of the amplicons, the PCR products were purified by standard protocol and Sanger sequenced using the BigDye Terminator v3.1 Cycle Sequencing Kit (ABI 3730 Applied Biosystem, USA) for the verification of candidate variants. The *STAG1* gene primers used for PCR amplification were as follows: forward 5′-TTTATCCAGTGTTCAGGA-3′, reverse 5′-AGGGTACTTGTATGCCTAA-3′. The identification of variants was performed using Chromas software (version2.2.6, Technelysium, Australia).

### Construction of the *STAG1* variant *in vitro* and transfection

Mutant *STAG1* gene pcDNA3.1(+)-FLAG-*STAG1*-mut and control pcDNA3.1(+)-FLAG-*STAG1*-WT mammalian expression vectors were constructed and purchased from Wuhan Biorun Biosciences Co., Ltd. Human embryonic kidney (HEK)-293T cells were transfected with expression vectors in two tubes (one for the mutant expression vectors and one for the wild-type expression vectors). The constructed expression vectors were verified using Sanger sequencing. Then, Six hours after the transfection, we gently removed the lipofectamine-containing medium, replaced the medium with Dulbecco's modified Eagle medium (DMEM) containing 5% fetal bovine serum (FBS), and incubated the slide at 37 ℃ in a 5% CO_2_ incubator until day 2. On day 3, the samples were collected and used in a reverse transcription-polymerase chain reaction (RT-PCR) to quantify the *STAG1* mRNA expression and a Western blot (WB) assay to quantify the STAG1 protein expression. Each cell line underwent three independent replicates of the RT-PCR assay. The RT-PCR and Western blotting experiments were conducted in accordance with the manufacturer's instructions.

### Reverse transcription-quantitative polymerase chain reaction (RT-qPCR)

After transfecting HEK293T cells with wild-type (*STAG1*-WT) and mutant (*STAG1*-mut) expression vectors for 24 h, we centrifuged the transfected cells to collect them, and then extract the total RNA from the cells using the Trizol method. Total RNA concentration and quality were checked using a NanoDrop 2000 spectrophotometer (Thermo Fisher Scientific, USA). Complementary DNA (cDNA) was synthesized using NovoScript® Plus All-in-one 1st Strand cDNA Synthesis SuperMix (Novoprotein, China) following the manufacturer's instructions. The cDNA concentration was about 1,200 ng/μl. The total reaction system was 20 µl, and the total RNA template was 500 ng. Quantitative PCR was performed using the Maxima SYBR Green/ROX qPCR Master Mix (2×) (Thermo Fisher Scientific, USA). Relative *STAG1* mRNA expression levels were determined using 2^−*ΔΔ*Ct^ method, and the *GAPDH* gene was used as the reference gene. The qRT-PCR reaction was performed in triplicate, and mean values and standard deviations were calculated from the obtained data. The primers used for PCR amplification of *STAG1* gene were as follows: forward 5′-AGAATTTGATGAGGACAGTGGTGA-3′, reverse 5′-TCAGGACTCCAATAAATTCACAAAA-3′.

### Western blot (WB)

After transfecting HEK293T cells with wild-type (*STAG1*-WT) and mutant (*STAG1*-mut) expression vectors for 24 h, western blot was performed using standard protocol. Equal amounts of protein were resolved by sodium dodecyl sulfate-polyacrylamide gel electrophoresis (SDS-PAGE) and subsequently transferred onto polyvinylidene fluoride (PVDF) membranes. Membranes were blocked with 5% non-fat milk in a tris buffered saline with tween-20 (TBST) solution for 2 h at room temperature. Anti-Flag (at a dilution of 1:1,000) and anti-glyceraldehyde phosphate dehydrogenase (anti-GAPDH) dilutions (at a dilution of 1:1,000) were added respectively and incubated at 4 ℃ overnight. Horseradish peroxidase (HRP)-labeled secondary antibodies were added and incubated at room temperature for 1 h. GAPDH served as the loading control and internal reference protein. The loading amount of WB protein was 10 μg/well. Blot bands were quantified by densitometry using ImageJ software (ImageJ2). Protein levels were normalized to GAPDH. The relative STAG1 protein expression level is quantified by the fold change (FC) in the ratio of the grayscale between the STAG1 protein band and the internal reference protein band.

### Statistical analysis

All data statistical comparisons between two groups (*STAG1*-WT and *STAG1*-mut) were evaluated by Student's *t*-test using Prism software (GraphPad Prism 10.5.0). *P* < 0.05 was considered statistically significant.

### Literature review

We searched the PubMed database using “*STAG1* gene” as keywords. The search time was from the establishment of the databases to February 31, 2025. We reviewed *STAG1*-related cases with availability of clinical data including relevant genetic testing results, prenatal/postnatal manifestations and pregnancy outcomes.

## Results

### Genetic analysis and confirmation

**Figure 1:**
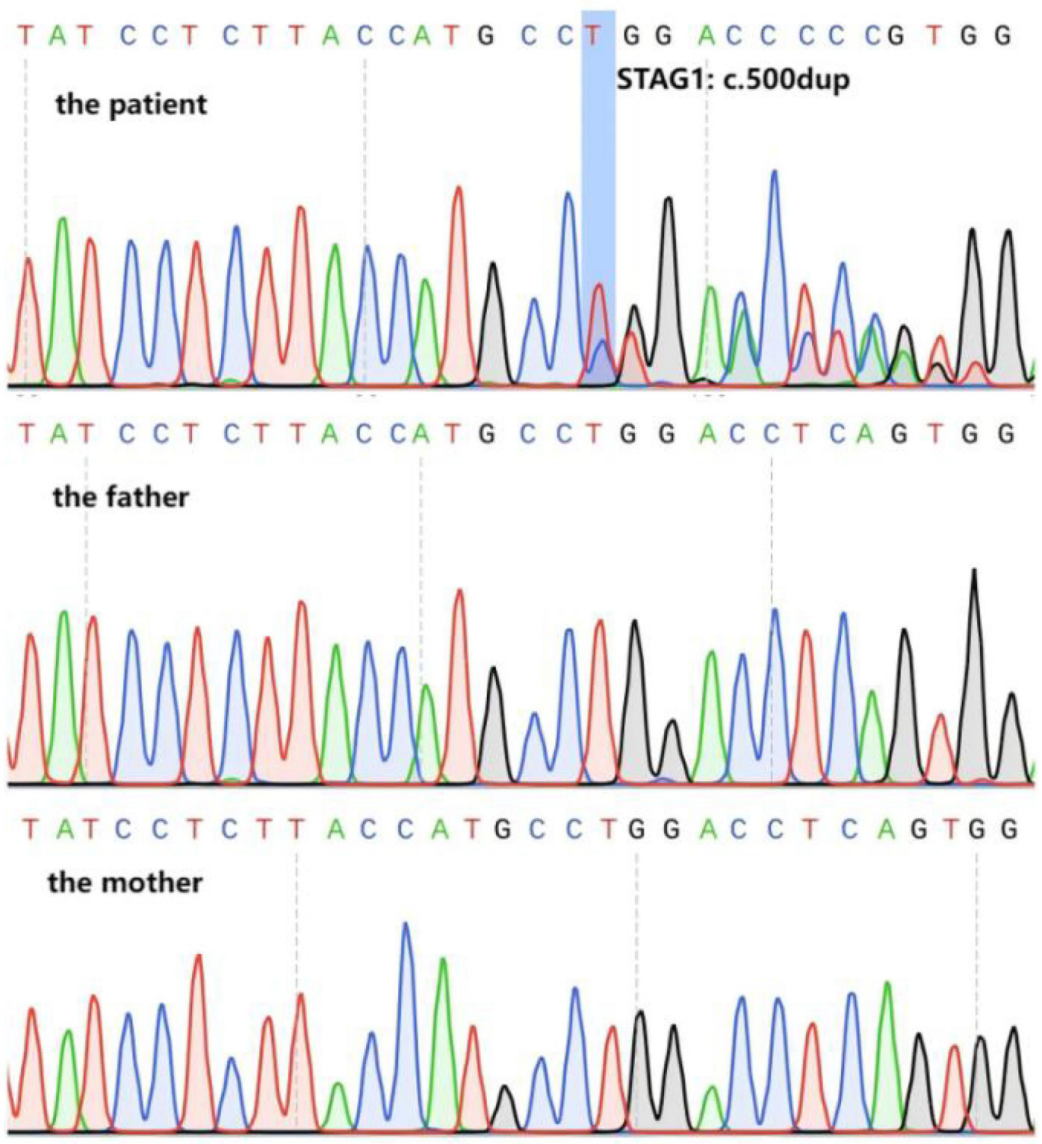
Variant verification of Sanger sequencing. Sanger sequencing confirmed that the patient carried the heterozygous frameshift variant in *STAG1* gene (NM_005862.3) c.500dup (marked by the blue box), the parents were normal.

Trio-WES identified a novel heterozygous frameshift variant in the *STAG1* gene (NM_005862.3) c.500dup (p.Gly168TrpfsTer13). This variant was further verified by Sanger sequencing, confirming that it was a *de novo* variant (Figure 1). The allele frequency of this heterozygous variant has not been registered in population databases (1,000 Genomes Project, gnomAD, and dbSNP) or reported in disease databases (ClinVar, HGMD, OMIM) (PM2_Supporting). This patient was the only *de novo* occurrence of this variant (PS2_Supporting). The probability of being LoF intolerant (pLI) value of *STAG1* gene in the gnomAD v4.1.0 was 1.0, which indicated that *STAG1* gene was extremely intolerant to loss-of-function variants (pLI > 0.9). The frameshift *STAG1* gene variant was a presumed LoF variant, LoF was a putative mechanism of *STAG1*-related disease (PVS1). As per the interpretation guidelines by ACMG (2), this novel variant was classified as “likely pathogenic”. We have submitted this variant to a public database [Leiden Open Variation Database (LOVD)], and the accession number can be found at the following URL: https://databases.lovd.nl/shared/individuals/00464708.

### Functional analysis of the frameshift variant

**Figure 2:**
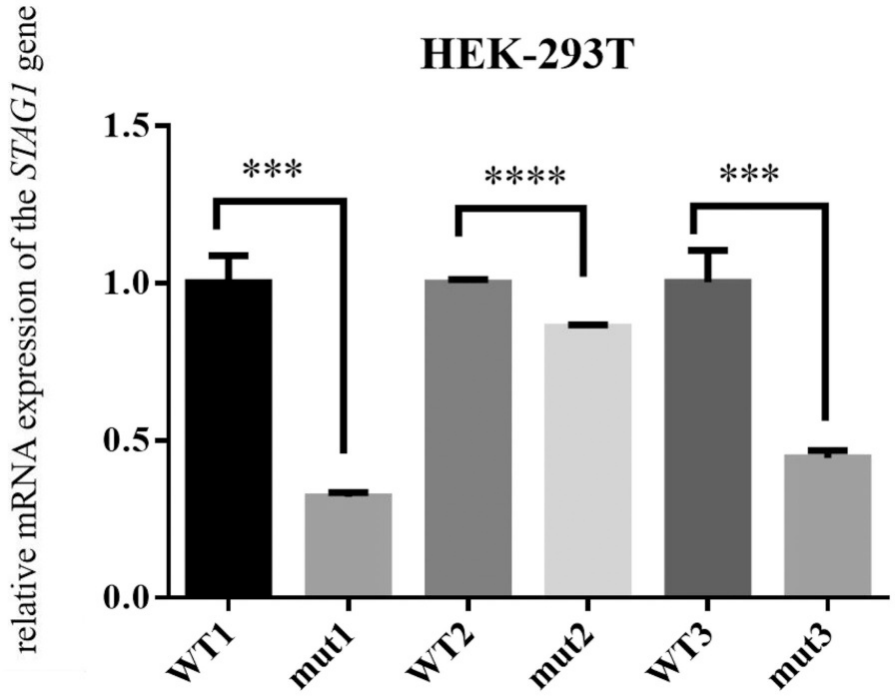
The relative mRNA expression of the *STAG1* gene. The results of RT-PCR analysis showed that the mRNA expression of mutant *STAG1* cell lines was significantly reduced compared to the *STAG1*-WT cell lines. Each cell line underwent three independent replicates of the RT-PCR assay, The difference between *STAG1*-WT and *STAG1*-mut was evaluated by Student's *t*-test. ***P < 0.001.

**Figure 3:**
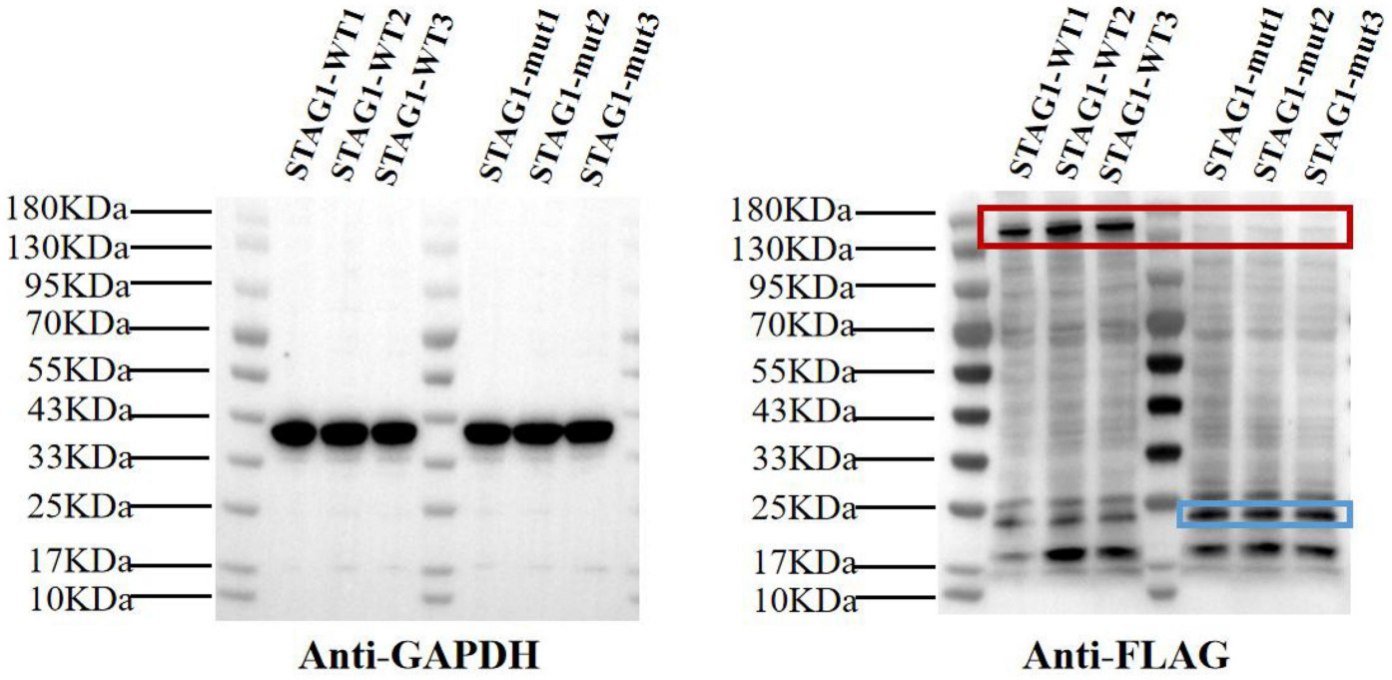
Western blot analysis of STAG1 protein. The results of WB analysis showed that mutant *STAG1* cell lines could not produce the normal STAG1 protein compared to the *STAG1*-WT cell lines (marked by the red box). The molecular mass of normal STAG1 (NM_005862.3) was about 144 kDa. The theoretical molecular mass of the truncated STAG1 protein was approximately 22.9 kDa (marked by the blue box). Each cell line underwent three independent replicates of WB analysis.

**Figure 4:**
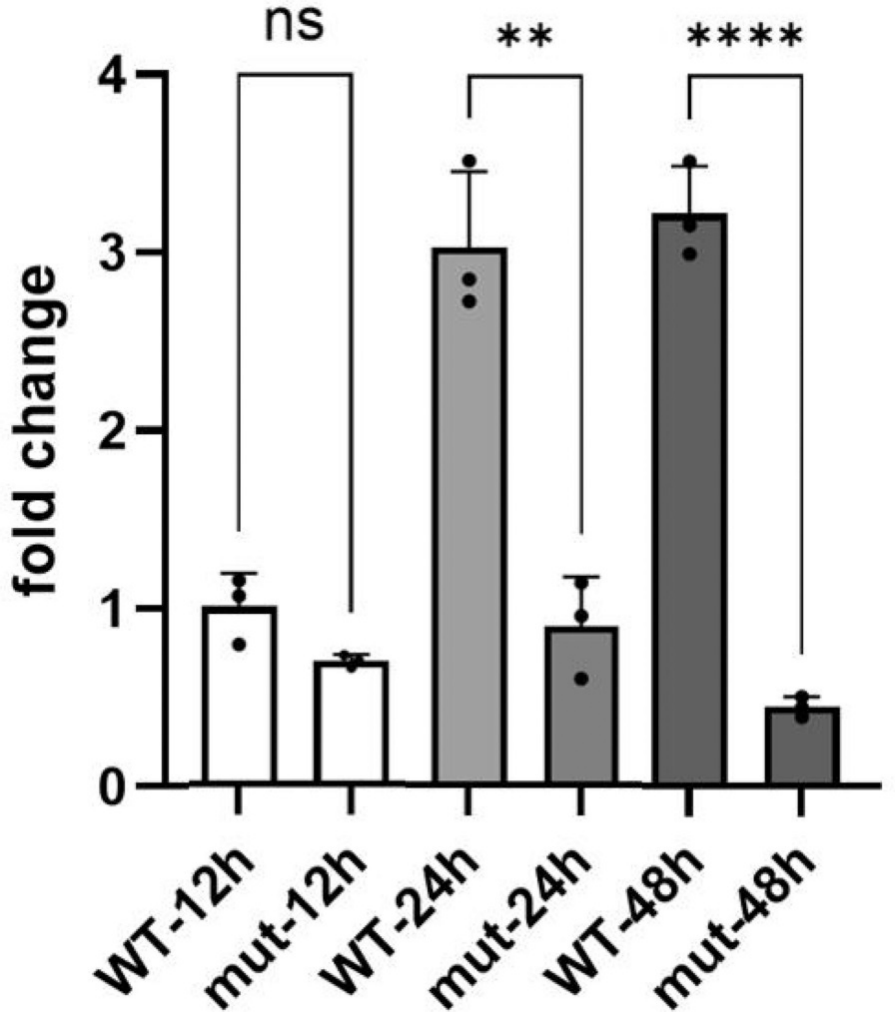
The relative STAG1 protein expression. The relative STAG1 protein expression was quantified by the fold change (FC). WB analysis indicated that the relative expression level of STAG1 protein in the *STAG1*-mut group was significantly lower than that in the *STAG1*-WT group. From 12 to 24 h, FC showed a slow upward trend (increasing from 0.69 to 0.90), while from 24 to 48 h, it exhibited a more significant downward trend (decreasing from 0.90 to 0.45). Each cell line underwent three independent replicates of WB analysis. The difference between *STAG1*-WT and *STAG1*-mut was evaluated by Student's *t*-test. **P < 0.01, ***P < 0.001, ns: not significant.

To evaluate the effect of the frameshift *STAG1* variant, our functional studies revealed that this *STAG1* variant, c.500dup, markedly decreased mRNA expression of *STAG1* gene (Figure 2), leading to a lack of normal STAG1 protein (Figures 3, 4), compared to the *STAG1*-WT cell lines.

## Discussion

The *STAG1* gene (OMIM*604358), known as “stromal antigen 1 (*SA1*)”, is located on chromosome 3q22.3. The transcript of *STAG1* (NM_005862.3) has 34 exons with the transcript length of 6,062 base pairs. Cohesin subunit SA-1 (STAG1 protein) also named as SCC3 homolog 1, is a 1258 amino acid protein (UniProt database accession Q8WVM7), which belongs to the SCC3 family (3). STAG1 is a subunit component of the cohesin complex (a ring-shaped structure is composed of four subunits: SMC1A, SMC3, RAD21/REC8, STAG1/2/3) required for sister chromatids cohesion along the length of a chromosome from DNA replication through prophase and prometaphase (4). At anaphase, the cohesin complex is cleaved and dissociates from chromatin, allowing sister chromatids to segregate (5). A spectrum of human developmental syndromes (such as Cornelia de Lange syndrome, Roberts-SC phocomelia syndrome, Warsaw breakage syndrome, chronic atrial and intestinal dysrhythmia syndrome, CHOPS syndrome, and alpha-thalassemia/impaired intellectual development syndrome), collectively termed “cohesinopathies”, refers to disorders resulting from variants in genes encoding the cohesin complex and its regulators (such as NIPBL, ESCO2, ANKRD11, HDAC8, DDX11, SGO1, AFF4, ATRX and others) (6). *STAG1*-related MRD47 (OMIM#617635) can also be classified as one of the cohesinopathies.

The *STAG1*-related cohesinopathy has been reported in seven studies, encompassing a total of 28 patients aged 0 to 29 years. We retrospectively reviewed a total of 18 cases with heterozygous missense/frameshift/nonsense *STAG1* variants (SNVs/indels) (1, 7–12) (Table 1). All these *STAG1* SNVs/indels were *de novo*. Among them, 2 were nonsense variants, 4 were frameshift variants, and 12 were missense variants. An additional 10 patients with copy-number deletions or intragenic deletions affecting the *STAG1* gene [regarded as “structural variations (SVs)”] have been reported, three of whom lacked detailed clinical documentation (1, 7). The most prevalent shared characteristics observed in these 25 patients included developmental delay/intellectual disability (DD/ID) (100%, 25/25), speech delay (100%, 25/25), and dysmorphic facial features (92%, 23/25). The earliest reported age of first spoken words among patients in the literature was 18 months, while the latest was 15 years. In our case, the patient remained non-verbal at 3 years of age, and the onset of language function recovery could not be determined. Other common clinical phenotypes of MRD47 included autism spectrum disorder (32%, 8/25) and epilepsy (32%, 8/25). During the evaluation period, this patient did not exhibit any signs of autistic behavior or seizure. Other uncommon clinical phenotypes of MRD47 included congenital heart disease (2/25), hearing loss (3/25), visual impairment (2/25), scoliosis (2/25), cryptorchidism (4/25), fifth finger clinodactyly (2/15), and syndactyly (2/15). None of these phenotypic features were observed in the present case. It has been reported that the *STAG1*-related clinical manifestations overlapped with the phenotype of Cornelia de Lange syndrome (12). The study by Lehalle et al. suggested that there was no notable disparity in clinical manifestations caused by *STAG1* deletions or *STAG1* variants apart from microcephaly (4/7 cases with SVs of *STAG1* had microcephaly, whereas 10 cases with *STAG1* SNVs/indels didn't have microcephaly) (1). Although the *STAG1*-related clinical manifestations showed the low specificity and clinical diversity, various degrees of DD/ID have been found in all *STAG1*-related cases. At the last clinical evaluation, our case didn't show facial dysmorphisms, epilepsy, autism, limb anomalies or behavioral anomalies. Among these cases with reported prenatal examination records (a total of 16 cases), we found the pregnancies of 14/16 cases (including our case) with *STAG1* SNVs/indels were uneventful, other two cases presented with abnormal genitalia and intrauterine growth retardation (IUGR). Therefore, it is warranted to further deliberate on the necessity of prenatal diagnosis for fetuses in low-risk pregnancies. Strikingly, the pregnancies of 66.7% cases (4/6) with SVs of *STAG1* were abnormal (such as IUGR, increased nuchal translucency, hydramnios, heart defects) (1). It can be inferred that the majority of MRD47 fetuses could not be diagnosed prenatally and are only identified postnatally. Consequently, it is worth further deliberation whether prenatal whole-exome sequencing is warranted in low-risk pregnancies.

**Table 1:** Summary of the clinical phenotypes of children with previously reported *STAG1* gene variants.

STAG1 variants (NM_005862.2)	Prenatal presentations	Primary postnatal presentations	Reference
c.641A>G (p.Gln214Arg)	Unremarkable	9 years and 9 months old, male, ID, speech delay, mild hearing loss, abnormal facial features, OFC on −1SD	(1)
c.1433A>C (p.His478Pro)	Unremarkable	29 years old, male, infantile febrile seizures, ID, speech delay, abnormal facial features, OFC on −1SD, scoliosis	(1)
c.646A>G (p.Arg216Gly)	Unremarkable	5 years 9 months old, female, feeding difficulties, DD, speech delay, abnormal facial features, OFC on −1.5 SD	(1)
c.1118G>A (p.Arg373Gln)	Unremarkable	2.5 years old, male, DD, hypotonia, feeding difficulties, speech delay, febrile seizures, sleep disturbance, subtle facial changes	(1)
c.1460_1464dup (p.Trp489Valfs*10)	Unremarkable	8 years old, female, ID, speech delay, autistic features	(1)
c.659A>G (p.His220Arg)	Unremarkable	8 years old, female, feeding difficulties, DD/ID, speech delay, autistic disorder, complex partial seizures, subtle facial features	(1)
c.997A>C (p.Lys333Gln)	Abnormal genitalia	3 years old, male, neonatal hypotonia, feeding difficulties, DD, speech delay, abnormal facial features, OFC on −0.5 SD, cryptorchidism	(1)
c.2936A>G (p.Lys979Arg)	Unremarkable	4 years old, female, feeding difficulties, DD, speech delay, subtle facial features	(1)
c.1052T>G (p.Leu351Trp)	Unremarkable	15 years old, male, feeding difficulties, DD/ID, speech delay, no facial features	(1)
c.1736 (p.Ser580Valfs*21)	Unremarkable	3 years old, female, DD/ID, speech delay, partial seizures, autistic features, subtle facial features	(1)
c.2009_2012del (p.N670Ifs*25)	Unremarkable	11 years old, female, ID, speech delay, autistic disorder, abnormal facial features, syndactyly, strabismus, precocious puberty, muscle fatigue	(7)
c.1129C>T (p.Arg377Cys)	NA	4 years old, male, DD, seizures during infancy, short stature, hypotonia, speech delay, abnormal facial features, microcephaly, congenital heart defect, behavioral problems, cryptorchidism, horseshoe kidney, clinodactyly, syndactyly, ectopic posterior pituitary	(7)
c.2769_2770del (p.Ile924Serfs*8)	Unremarkable	5 years old, female, DD, speech delay, abnormal facial features, micrognathia, microcephaly, clinodactyly, pectus excavatum, juvenile idiopathic arthriti	(8)
c.901C>T (p.Arg301Cys)	Intrauterine growth retardation	3 years old, male, DD, feeding difficulties, speech delay, short stature, abnormal facial features, hypoplasia of the mandibl, cryptorchidism, bilateral microtia, bilateral hearing loss	(9)
c.1279G>A (p.Val427Ile)	Unremarkable	9 years old, monozygotic twin Ⅰ, male, prematurity (week 33), ID, speech delay, bilateral flat foot, hyperlaxity and obesity, abnormal facial features	(10)
c.1279G>A (p.Val427Ile)	Unremarkable	9 years old, monozygotic twin Ⅱ, male, prematurity (week 33), ID, speech delay, bilateral flat foot, hyperlaxity and obesity, abnormal facial features, behavioral problems	(10)
c.1183C>T, (p.Arg395*)	Unremarkable	3 years old, female, DD, speech delay, abnormal facial features, brachycephaly, micrognathia, bilateral clubfoot, loss of vision, microphthalmia, strabismus	(11)
c.17T>G (p.Leu6*)	NA	DD/ID, short stature, abnormal facial features, hirsutis, information on age and sex were not available	(12)

ID, intellectual disability; DD, developmental delay; OFC, occipitofrontal circumference.

Based on these cases with frameshift/nonsense *STAG1* variants and copy-number deletions/intragenic deletions of *STAG1*, Lehalle et al. (1), Di Muro et al. (8) and Bregvadze et al. (11) postulated that a pathogenic mechanism known as haploinsufficiency (loss of one of two functional alleles) was caused by *STAG1* LoF variants, which may result in partial or complete knockdown of protein activity or product. Our functional studies *in vitro* of this variant have been performed, RT-PCR showed that the mRNA expression of mutant *STAG1* cell lines was extremely lower than wild-type mRNA (Figure 2). Theoretically, the heterozygous frameshift variant c.500dup produced a premature termination codon (PTC), leading to a truncated protein consisting of 180 amino acids.

The result of WB analysis showed mutant *STAG1* cell lines could not produce the normal STAG1 protein compared to the *STAG1*-WT cell lines (Figure 3). Based on the these results, it could be inferred that the heterozygous frameshift variant c.500dup resulted in a partial absence of the biologically relevant transcript of the *STAG1* gene (NM_005862.3) attributed to an mRNA quality-control mechanism of nonsense-mediated mRNA decay (NMD). NMD surveys newly synthesized mRNAs and degrades those that harbor a PTC (13), leading to complete absence of the normal STAG1 protein (Figure 4 showed that from 12 to 24 h, the level of the mutant protein progressively declined due to mutant mRNA decay, whereas the wild-type protein remained stable due to mutant mRNA decay). Thereby, this variant c.500dup prevented the production of normal STAG1 protein, which caused the *STAG1*-related cohesinopathy.

In conclusion, there was no significant difference in the clinical manifestations observed between patients with *STAG1* deletions and those with *STAG1* SNVs/indels, except for microcephaly and abnormal pregnancies. The *STAG1*-related cohesinopathy exhibited nonspecific characteristics and diverse presentations, but various degrees of ID/DD have been observed in all cases. Our functional studies have provided preliminary evidence confirming the haploinsufficiency of the *STAG1* gene as the underlying pathogenic mechanism of the *STAG1*-related cohesinopathy.

## Data Availability

The datasets presented in this article are not readily available because of ethical and privacy restrictions. Requests to access the datasets should be directed to the corresponding author.
